# Validity of self-reported and objectively measured sedentary behavior in pregnancy

**DOI:** 10.1186/s12884-020-2771-z

**Published:** 2020-02-11

**Authors:** Bethany Barone Gibbs, Joshua L. Paley, Melissa A. Jones, Kara M. Whitaker, Christopher P. Connolly, Janet M. Catov

**Affiliations:** 1 Department of Health and Physical Activity, Clinical and Translational Sciences, University of Pittsburgh, 32 Oak Hill Court, Room 220, Pittsburgh, PA 15261 USA; 2 Department of Clinical and Translational Sciences, University of Pittsburgh, Pittsburgh, USA; 3 Department of Health and Human Physiology, Department of Epidemiology, University of Iowa, Iowa City, USA; 4 Department of Educational Leadership, Sport Studies, and Educational/Counseling Psychology, Washington State University, Pullman, USA; 5 Department of Obstetrics, Gynecology, and Reproductive Sciences, Magee-Womens Hospital of the University of Pittsburgh Medical Center, Pittsburgh, USA

**Keywords:** activPAL, Accelerometer, Sedentary time, Trimester, Self-report questionnaire

## Abstract

**Background:**

Sedentary behavior (SED) is a potential risk factor for poor pregnancy outcomes. We evaluated the validity of several common and one new method to assess SED across three trimesters of pregnancy.

**Methods:**

This cohort study of pregnant women measured objective and self-reported SED each trimester via thigh-worn activPAL3 micro (criterion), waist-worn Actigraph GT3X, and self-report from the Pregnancy Physical Activity Questionnaire (PPAQ) and the de novo Sedentary Behavior Two Domain Questionnaire (SB2D). SED (hours per day) and percent time in SED (SED%) from activPAL were compared to GT3X, SB2D, and PPAQ using Pearson’s *r*, ICC, Bland-Altman analysis, and comparison of criterion SED and SED% across tertiles of alternative methods.

**Results:**

Fifty-eight women (mean age 31.5 ± 4.8 years; pre-pregnancy BMI 25.1 ± 5.6 kg/m^2^; 76% white) provided three trimesters of valid activPAL data. Compared to activPAL, GT3X had agreement ranging from *r = 0*.54–0.66 and ICC = 0.52–0.65. Bland-Altman plots revealed small mean differences and unpatterned errors, but wide limits of agreement (greater than ±2 h and ± 15%). The SB2D and PPAQ had *r* < 0.5 and ICC < 0.3 vs. activPAL SED, with lower agreement during the 2nd and 3rd trimesters, and performed poorly in Bland-Altman analyses. SED% from the modified SB2D performed best of the self-reported instruments with modest mean differences, r ranging from 0.55 to 0.60, and ICCs from 0.31–0.33; though, limits of agreement were greater than ±35%. Significant trends in activPAL SED were observed across increasing tertiles of SB2D SED in the 1st and 3rd trimesters (both *p* ≤ 0.001), but not the 2nd trimester (*p* = 0.425); and for PPAQ SED in the 1st and 2nd trimesters (both *p* < 0.05), but not the 3rd trimester (*p* = 0.158). AcitvPAL SED and SED% increased significantly across tertiles of GT3X SED and SED% as well as SB2D SED% (all *p-for-trend* ≤ 0.001*)*.

**Conclusions:**

Compared to activPAL, waist-worn GT3X produced moderate agreement, though similar mean estimates of SED across pregnancy. Self-report questionnaires had large absolute error and wide limits of agreement for SED hr./day; SB2D measurement of SED% was the best self-report method. These data suggest activPAL be used to measure SED when possible, followed by GT3X, and – when necessary – SB2D assessing SED% in pregnancy.

**Trial registration:**

www.clinicaltrials.gov NCT03084302 on 3/20/2017.

## Introduction

Sedentary behavior, defined as waking behavior in a seated, lying, or reclining posture and at low intensity (< 1.5 metabolic equivalents [METs]) [1], is an emerging risk factor for outcomes such as weight gain [2], cardiometabolic disease [3, 4], and depression [5]. Pregnancy is a biologically relevant period during which adverse outcomes, such as excessive gestational weight gain, gestational hypertension, and gestational diabetes, can manifest [6]. Though regular physical activity during pregnancy can protect against these outcomes [7], most pregnant women do not achieve recommended levels [8, 9]. Preliminary data suggests that pregnant women also engage in high levels of sedentary behavior [8, 10], independent of engagement in moderate-to-vigorous intensity physical activity. Thus, sedentary behavior reduction may be a distinct and potentially feasible behavioral target to improve pregnancy health.

Despite the possibility that sedentary behavior is a novel risk factor during pregnancy, there is a dearth of high quality research evaluating sedentary behavior patterns across gestation and associated outcomes in pregnant women. A recent systematic review concluded that, though more than 25 studies have evaluated sedentary behavior in pregnancy, substantial heterogeneity in methodology limits the ability to synthesize findings [11]. The primary recommendation of this review was that studies using ‘robust methodology for quantifying sedentary behavior’ are most needed [11]. This highlights an overall challenge in sedentary behavior research where definitions and best practice assessment methodology have only recently emerged [1, 12].

Existing studies of sedentary behavior in pregnant women have used both self-report questionnaires and objective monitors. These have commonly included self-report by the sedentary behavior subscale on the Pregnancy Physical Activity Questionnaire (PPAQ) [13, 14] or an accelerometer [10, 15–17]. However, these methodologies have distinct limitations as compared to the current best practice of a thigh-mounted inclinometer and accelerometer (e.g., activPAL), which has been used in fewer pregnancy studies [11, 18]. The activPAL is preferred due to its capability of capturing both the postural (seated/reclining/lying) and intensity (< 1.5 METs) aspects of the current consensus definition of sedentary behavior [1]. Daily participation in sedentary behavior is known to be poorly estimated by self-report instruments in non-pregnant populations [19], a phenomenon that may reflect its high frequency and intermittency throughout the day. While objective monitoring is thus preferred to quantify total sedentary time [20], the commonly used method of waist- or wrist-worn accelerometry (e.g., Actigraph) is limited in that it only measures the absence of movement (i.e., stationary behavior) rather than the definition of sedentary behavior that includes posture and intensity [1]. This may be further compromised in the later stages of pregnancy where the standard position of waist-worn accelerometry must be adjusted [21].

Unfortunately, among pregnant women, the ability of self-reported or accelerometer-measured sedentary behavior as compared to activPAL to estimate daily duration or rank women by level of sedentary behavior is currently unknown. This is important for both interpretation of available data and also for planning of future research relating sedentary behavior to maternal-fetal outcomes. To address this gap, we used data from an ongoing cohort study measuring objective sedentary behavior in pregnant women across three trimesters. We aimed to evaluate agreement between accelerometer-measured sedentary behavior as well as several self-report instruments as compared to best practice assessment with a thigh-mounted activPAL monitor in each trimester of pregnancy.

## Methods

### Participants and setting

This study uses data from a subsample of participants enrolled in the Monitoring Movement and Health Study (MoM Health), a longitudinal cohort study characterizing sedentary behavior, physical activity, and pregnancy health outcomes across each trimester of pregnancy (clinicaltrials.gov identifier: NCT03084302). Pregnant women were recruited for the parent study using fliers at and around obstetrics and gynecology practices, word-of-mouth, a University-based research registry, and emails to University of Pittsburgh employees. Women were eligible to participate in the MoM Health study if they were: less than 14 weeks pregnant, planning to have prenatal care and deliver at a University of Pittsburgh Medical Center facility, and able to attend all study visits. Women were excluded if they had severely limited mobility (defined as unable to walk ½ mile or up 2 flights of steps), were currently taking medication to control blood pressure or diabetes, had a severe medical condition (e.g., chronic obstructive pulmonary disease, renal disease), or if they were currently participating in another research study intending to modify their lifestyle behavior. All procedures were approved by the University of Pittsburgh Institutional Review Board and all participants provided written informed consent prior to participating in the study.

MoM Health Study participants attended three study visits: first trimester (between 8 and 13 weeks); second trimester (between 20 and 22 weeks); and third trimester (between 32 and 34 weeks). To be included in the current validation study, participants were required to have completed all three study visits with valid criterion sedentary behavior measurement by the activPAL3 micro (described below). Of the first 65 enrolled participants that completed study visits during all three trimesters, 58 women met this criteria. Seven women were excluded due to device failure (*n* = 5) or lost monitors (*n* = 2) at one of the three visits.

### Measures

#### Demographics and clinical measures

Participant characteristics were self-reported on standard questionnaires. Pre-pregnancy weight was abstracted from participant medical records and height measured by stadiometer with shoes removed at the first trimester assessment visit. These were used to calculate pre-pregnancy body mass index (BMI) as kg/m^2^.

#### activPAL3 micro (criterion)

Measurement of sedentary behavior used the activPAL3 micro (PALtechnologies, Glastgow, Scotland) thigh-mounted accelerometer and inclinometer as well as published protocol recommendations [1, 12, 22]. During each visit, participants first received verbal and written instructions then self-applied the monitor to the anterior thigh using a provided Tegaderm® dressing. The research personnel then confirmed correct placement. Participants were instructed to wear the monitor 24 h per day, for 7 complete days, with removal only for swimming. Seven additional Tegaderm® dressings were provided and participants were instructed that they could change dressings and alternate legs as needed if the dressing came loose or the skin underneath became irritated. During monitor wear, participants completed a diary that reported time awoke in the morning, time went to sleep, naps, and any removal of the device. Event data from the activPAL were exported, cleaned, and reduced by trained research personnel using standardized procedures that combined diary and objective data to identify waking wear periods across the monitoring interval [12]. For each wear day, daily sedentary time (SED) in hours per day was calculated as the sum of all SED intervals during waking hours. A minimum of 4 days with at least 10 h per day of monitoring was required to be considered valid. Estimates of daily SED and wear time (hours per day) as well as percentage of time sedentary (SED% = SED divided by wear time) were averaged over valid days.

#### Actigraph GT3X

Participants were instructed to wear the Actigraph GT3X accelerometer (Actigraph, Pensacola, FL) on an elastic belt fastened snugly to their torso, directly over the right side of their right kneecap during all waking hours, with removal while sleeping and during water activities (bathing or swimming). The GT3X was worn concurrently with the activPAL over 7 complete days. Because of changing anthropometry across pregnancy, and from previous research by the study team [23], pictures were provided to aid in correct device placement (with elastic belt below belly, as needed). Accelerometer data (60-s epochs) were exported and reduced using ActiLife Software v6.13.3. Nonwear time was identified using an automated protocol of any period with at least 60 consecutive minutes of 0 counts per minute (cpm), with an allowance for 2 min of < 100 cpm [24]. SED was defined as any 60-s epoch with < 100 cpm during valid wear time [24, 25]. A minimum of 4 days with at least 10 h per day of monitoring was required to be included in analysis [24]. Daily estimates of SED, wear time (hours per day), and %SED (SED divided by wear time) were averaged over valid days. Of the 58 women meeting criteria for valid activPAL data at each assessment visit, 57, 56, and 51 women had valid GT3X data at the 1st, 2nd, and 3rd trimesters respectively. Data were missing due to insufficient wear time (*n* = 7) or device failures (*n* = 3).

#### Self-report

Sedentary behavior was assessed using two self-report methods. First, the sedentary behavior subscale from the Pregnancy Physical Activity Questionnaire (PPAQ) [26] was included as it is a validated and commonly used instrument to assess time spent in participating in a variety household/caregiving, occupational, sports/exercise, and transportation activities among pregnant women [27–29]. The PPAQ estimates SED in hours per day by summing duration x intensity for questions 12, 13, 30, and 31 (if open-ended questions are < 1.5 METs) [26]. Reflecting the 2017 consensus definition of sedentary behavior [1] published after the PPAQ sedentary behavior subscale was published in 2004, we additionally summed responses to PPAQ questions 11 (sitting using a computer or writing while not at work), 22 (driving or riding in a car or bus), and 32 (sitting at work or in class) as recommended by the DAPA Measurement Toolkit (*https:/dapa-toolkit.mrc.ac.uk/pdf/pa/PPAQ_instructions_1.pdf*). Of note, we slightly modified the scoring algorithm which includes multiplying durations by intensity for each item on the sedentary behavior subscale. Rather, we chose to only sum durations as this was most comparable to the duration estimate from our criterion measure. When we repeated analyses using the published scoring algorithm and comparing PPAQ SED MET-hours per day to activPAL SED hours per day, results were either less or similarly correlated to the criterion measure (data not shown). One participant missed two SED questions on the PPAQ during the 2nd trimester and was not included for comparisons between activPAL and PPAQ for that visit (*n* = 57).

Second, at the beginning of the MoM Health Study, two short de novo instruments were developed to assess SED with the purpose of validation among pregnant women (hereafter referred to as the Sedentary Behavior Two Domain Questionnaire, SB2D). We used the language from the sedentary behavior question from the Global Physical Activity Questionnaire (GPAQ), [30] which is commonly used to assess sedentary behavior [31, 32] and which we found to be superior to a multi-domain sedentary behavior questionnaire in a previous validation study among young adults with a similar mean age to our population [33]. Next, we modified the question to capture SED (hours per day) separately on work (if applicable) and non-work days, similar to another sedentary behavior questionnaire by Whitfield, et al., [34] as employment is an important determinant of sedentary behavior in adult populations [35]. Last, in agreement with a recent systematic review of sedentary behavior questionnaire taxonomy that concluded SED% rather than absolute SED is recommended for population surveillance [36], we repeated the two-item instrument using Likert-type answers taken from the Canadian Fitness Survey [37] (response options were: almost none of the time, ¼ of the time, ½ of the time, ¾ of the time, almost all of the time). To combine self-reported SED and SED% on work and non-work days, estimates were scaled as follows: full-time employment: (5/7) x workday estimate + (2/7) x non-workday estimate; part-time employment: (2.5/7) x workday estimate + (4.5/7) x non-workday estimate; not employed: (7/7) x non-workday estimate (see Additional file 1: for SB2D questions and scoring). Though not specific to pregnant populations, these questions were included for evaluation as potentially simple instruments to be used in future research.

### Statistical analyses

All analyses were conducted using Stata version 14 (StataCorp, College Station, TX). Demographic and clinical measures were summarized using means and percentages. SED and SED% from alternative assessment methods (GT3X and self-report questionnaires) were compared to the criterion measure (activPAL) using the Bland-Altman method [38] at each trimester. Pearson’s correlations (*r*), intraclass correlation coefficients (ICC), and reporting of criterion-measured sedentary time distribution across tertiles evaluated the ability of alternative assessment methods to correctly rank women by their participation in sedentary behavior.

## Results

Approximately three fourths of the study population were white and had at least a bachelor’s degree (Table 1). Prior to pregnancy, women had an average BMI of 25.1 (SD 5.6) kg/m^2^. Women were assessed, on average, at gestational weeks 11.9 (SD 1.8), 21.2 (SD 0.9), and 33.3 (SD 0.9) (Table 1). By activPAL (the criterion method), women spent approximately 9.5 h per day and 63% of their waking time in sedentary behavior in each of the three trimesters (Table 2).

**Table 1:** Participant Characteristics (*n* = 58)

	mean (SD) or n (%)
Age	31.5 (4.8)
Race	
White	44 (76%)
Black	7 (12%)
Asian	3 (5%)
Multiracial	4 (7%)
Education	
< High school	1 (2%)
High school graduate	15 (26%)
College graduate	14 (24%)
Post-graduate degree	28 (48%)
Pre-pregnancy Body Mass Index, kg/m^2^	25.1 (5.6)
Gestational Age, week	
Visit 1 (1st trimester, 8–13 weeks)	11.9 (1.8)
Visit 2 (2nd trimester, 20–22 weeks)	21.2 (0.9)
Visit 3 (3rd trimester, 32–34 weeks)	33.3 (0.9)

**Table 2:** Sedentary behavior (SED) in hr./day, percent time in SED (SED%), correlations (*r*), and Intraclass Correlation Coefficients (ICC) between activPAL and GT3X, the Sedentary Behavior Two Domain Questionnaire (SB2D), and the Pregnancy Physical Activity Questionnaire (PPAQ)

	1st Trimester (< 14 weeks)			2nd Trimester (20–22 weeks)			3rd Trimester (32–34 weeks)		
	mean (SD)	*r*	ICC	mean (SD)	*r*	ICC	mean (SD)	*r*	ICC
SED, hrs per day									
*activPAL*	9.63 (1.55)	1.0	1.0	9.54 (1.24)	1.0	1.0	9.43 (1.29)	1.0	1.0
*GT3X* ^*a*^	9.50 (1.38)	0.62	0.61	9.08 (1.39)	0.58	0.55	9.08 (1.28)	0.54	0.52
*SB2D*	5.75 (2.20)	0.48	0.15	5.89 (2.51)	0.26	0.08	6.12 (3.06)	0.37	0.13
*PPAQ*^*b*^	8.17 (3.27)	0.42	0.28	8.49 (3.39)	0.24	0.14	7.95 (2.78)	0.28	0.18
SED%									
*activPAL*	0.64 (0.10)	1.0	1.0	0.63 (0.09)	1.0	1.0	0.63 (0.09)	1.0	1.0
*GT3X*^*a*^	0.66 (0.08)	0.66	0.62	0.64 (0.08)	0.64	0.63	0.64 (0.08)	0.66	0.65
*SB2D – Likert*	0.57 (0.21)	0.44	0.32	0.55 (0.24)	0.52	0.31	0.60 (0.21)	0.45	0.33
*PPAQ*		n/a			n/a			n/a	

^a^Sample sizes were *n* = 57 for 1st trimester; *n* = 56 for 2nd trimester; and *n* = 51 for 3rd trimester

^b^ Sample sizes was *n* = 57 for the 2nd trimester

### Comparison of Actigraph GT3X to activPAL3 micro

**Fig. 1:**
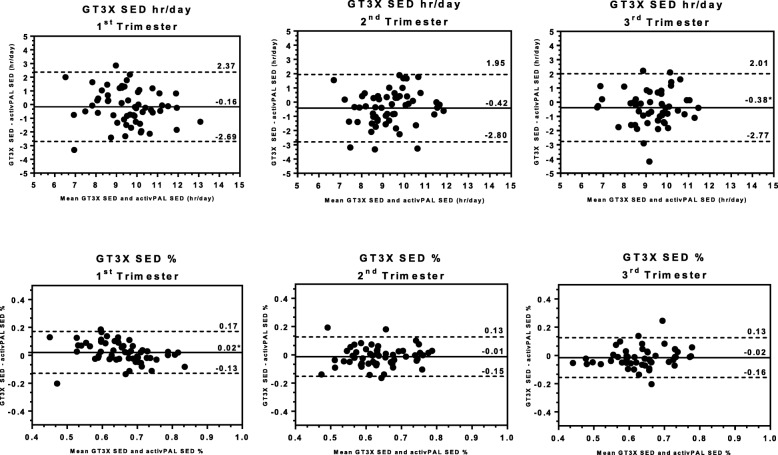
Bland-Altman Plots comparing Sedentary Behavior assessed by Waist-worn monitor (Actigraph GT3X) versus Thigh-worn monitor (activPAL3 micro) across Pregnancy Trimesters

Average SED was similar between the GT3X and activPAL in all trimesters (Table 2; Fig. 1). Correlations and ICCs of the GT3X with the activPAL ranged from 0.54 to 0.62; ICC ranged from 0.52 to 0.61 (Table 2). Bland-Altman plots from each trimester (first row, Fig. 1) revealed minimal systematic bias (underestimation by < 0.5 h per day), which was nonsignificant in the 1st and 2nd trimester with *p* < 0.05 in the 3rd trimester. No discernable pattern of errors across values of SED was observed. However, limits of agreement ranged more than 2 hours in either direction. When women were separated into tertiles based on GT3X SED, criterion SED increased across tertiles in every trimester (*p-for- trend* ≤ 0.001; Table 3).

**Table 3:** Sedentary behavior from the activPAL3 across Tertiles of Sedentary Behavior from the GT3X, the Sedentary Behavior Two Domain Instrument (SB2D), and the Pregnancy Physical Activity Questionnaire (PPAQ)

	Tertile 1		Tertile 2		Tertile 3		*p-for-linear trend*
	mean	(SD)	mean	(SD)	mean	(SD)	
SED hr./day							
*GT3X* ^*a*^							
1st Trimester	8.58	(1.34)	9.84	(1.09)	10.54	(1.55)	< 0.001
2nd Trimester	8.69	(1.04)	9.59	(1.09)	10.27	(1.07)	< 0.001
3rd Trimester	8.65	(1.33)	9.66	(0.97)	10.07	(1.05)	0.001
*SB2Q*							
1st Trimester	8.85	(1.60)	9.47	(1.16)	10.69	(1.29)	< 0.001
2nd Trimester	9.38	(1.20)	9.56	(1.25)	9.69	(1.31)	0.425
3rd Trimester	8.84	(0.96)	9.34	(1.31)	10.14	(1.28)	0.001
*PPAQ*^*b*^							
1st Trimester	8.84	(1.45)	9.53	(0.99)	10.59	(1.63)	< 0.001
2nd Trimester	9.07	(1.40)	9.63	(1.01)	10.00	(1.14)	0.018
3rd Trimester	8.95	(1.12)	9.79	(1.02)	9.51	(1.68)	0.158
SED%							
*GT3X*^*a*^							
1st Trimester	0.58	(0.09)	0.64	(0.09)	0.71	(0.08)	< 0.001
2nd Trimester	0.58	(0.08)	0.62	(0.05)	0.70	(0.07)	< 0.001
3rd Trimester	0.57	(0.10)	0.62	(0.05)	0.70	(0.08)	< 0.001
*SB2D – Likert*							
1st Trimester	0.61	(0.09)	0.60	(0.08)	0.71	(0.09)	0.001
2nd Trimester	0.59	(0.07)	0.66	(0.08)	0.69	(0.09)	< 0.001
3rd Trimester	0.58	(0.07)	0.66	(0.08)	0.68	(0.12)	0.001

^a^Sample sizes were *n* = 57 for 1st trimester; *n* = 56 for 2nd trimester; and *n* = 51 for 3rd trimester

^b^ Sample sizes was *n* = 57 for the 2nd trimester

Average SED% was also similar in each trimester comparing the GT3X to the activPAL (Table 2). Similar to the results for SED, correlations and ICCs for the GT3X as compared to the activPAL for SED% ranged from 0.64 to 0.66; ICC ranged from 0.62 to 0.65 (Table 2). Bland-Altman plots (second row, Fig. 1) again found minimal systematic bias (≤ 2% per day), which was only statistically significant in the first trimester (*p* < 0.05), and no clear pattern of errors. Limits of agreement were approximately ±15% at each trimester. Criterion SED% increased across increasing tertiles of GT3X SED% for each trimester (*p-for-trend* < 0.001; Table 3).

### Comparison of self-report to activPAL3 micro

**Fig. 2:**
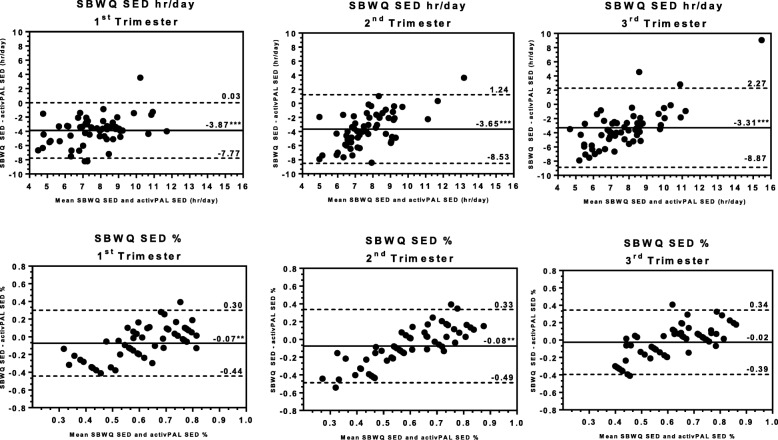
Bland-Altman Plots comparing Sedentary Behavior assessed by the Sedentary Behavior Two Domain Questionnaire (SB2D) versus Thigh-worn monitor (activPAL3 micro) across Pregnancy Trimesters

Average SED reported as hours per day on the SB2D underestimated time spent sedentary by more than 3 h across trimesters of pregnancy (Table 2; Fig. 2). Correlations ranaged from *r* = 0.26 to *r* = 0.48 and with ICCs < 0.20. From the Bland-Altman analyses, SED was significantly underestimated in every trimester (*p* < 0.001), limits of agreement were ± 4–5 h, and a pattern emerged in the 2nd the 3rd trimesters where SB2D tended to overestimate SED at low values and underestimate SED at high values (first row, Fig. 2). Tertile analyses (Table 3) revealed that criterion SED increased across tertiles of SB2D SED in the 1st and 3rd trimesters (*p*-*for-trend* ≤ 0.001), but not the 2nd (*p-for-trend* = 0.425).

Average SED% from the SB2D (with Likert responses) underestimated SED% between 2 and 8%. Correlations with the activPAL ranged from *r* = 0.44 to *r* = 0.52 across trimesters. Though higher than those for SED, ICCs between SB2D and activPAL for SED% were still low (ranged from 0.31 to 0.33; Table 2). Bland Altman analyses revealed systematic underestimation in the 1st and 2nd trimesters (*p* < 0.01), but not the 3rd (second row, Fig. 2). A similar pattern of error was observed where the SB2D tended to overestimate SED% at low values and underestimate SED% at high values. This resulted in wide limits of agreement that ranged from ±36 to ±41%. Criterion SED% increased across tertiles of SB2D SED% in each trimester (all *p-for -trends* < 0.001, Table 3).

**Fig. 3:**
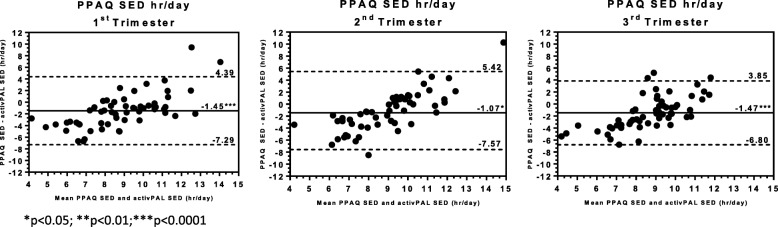
Bland-Altman Plots comparing Sedentary Behavior assessed by the Pregnancy Physical Activity Questionnaire versus Thigh-worn monitor (activPAL3 micro) across Pregnancy Trimesters

Average SED reported as hours per day on the PPAQ significantly underestimated sedentary time across trimesters by approximately 1–1.5 h (Table 2; Fig. 3). Correlations ranged from 0.24 to 0.42; ICC ranged from 0.14 to 0.28). Bland-Altman analysis again revealed a similar pattern of errors, with overestimation at low values of SED and underestimation at high values. Limits of agreement were approximately ±5.5 to 6.5 h per day. Criterion SED increased significantly across tertiles of PPAQ SED in the 1st and 2nd (*p-for-linear-trends* < 0.05) but not the 3rd trimesters (*p-for-trend* = 0.158).

As no total wear time or relative responses were available from the PPAQ, evaluation of the SED% from the PPAQ was not possible.

## Discussion

With the growing interest of sedentary behavior as a potential risk factor for adverse pregnancy outcomes [11], this study aimed to evaluate the validity of alternative methods versus the criterion activPAL3 micro to measure and rank women by level of sedentary behavior across three trimesters of pregnancy.

These findings make a contribution as the first study, to our knowledge, to evaluate the validity of sedentary behavior assessed by waist accelerometer versus the current best practice of a thigh-mounted activPAL during pregnancy. Our findings are comparable to similar validation research in non-pregnant adults. A recent validation study in 266 postmenopausal (non-pregnant) women found that waist-worn GT3X assessment resulted in very small differences in average SED (< 0.1 h per day) but wide limits of agreement (− 2.7 to 2.6 h per day), suggesting unbiased but poor individual-level agreement [15]. This comparison suggests that errors in SED measurement comparing the GT3X to an activPAL are likely similar in pregnant and non-pregnant populations. It is notable that, for SED, the GT3X significantly underestimated SED only in the 2nd and 3rd trimesters and correlations and ICCs decreased across pregnancy. However, this pattern was not observed for SED%. As such, it is possible that anatomical changes that occur across pregnancy may have increased the error in waist-worn GT3X SED estimated using validated cutpoints in the vertical axis. This possibility is supported by similar research finding that the accuracy of objective monitors for estimating energy expenditure differs across trimesters of pregnancy [21]. Since most of the handful of studies with objective measurement of sedentary behavior in pregnancy have used Actigraph accelerometers [11], our results are helpful when interpreting other study findings in that summary estimates are likely accurate but individual-level measurement error may attenuate associations with health outcomes [39]. Further, as interventions to decrease sedentary behavior would typically target behaviors in a seated posture such as time spent sitting/reclining watching television, sitting at a desk or table, or lying in bed using a smart phone or tablet, activPAL assessment should be the preferred method when individual precision is important (e.g., to test intervention or longitudinal effects on sedentary behavior). This may be especially important for interventions targeting the second half of pregnancy since imprecision may increase as pregnancy progresses.

We are also unaware of other studies comparing the PPAQ or other self-report measures to activPAL SED in pregnant women. One study used the GT3X as the criterion SED measure and found similar correlations for GPAQ (*r* = 0.4) or PPAQ (*r* = 0.3) across pregnancy [31]. The poor agreement of the PPAQ for measuring SED, especially in later trimesters, is disappointing as this questionnaire is common in pregnancy research studies. Yet, such agreement is comparable to other self-report versus objective measures of moderate-to-vigorous physical activity in pregnant women [40]. Further, the PPAQ importantly offers data on domain-specific SED, which can aid in designing interventions [19] and is not measured by the other methodologies evaluated in this analysis. Another important consideration is that we used an updated (DAPA Measurement Toolkit) formula for calculating SED from the PPAQ rather than the originally proposed algorithm. While the updated formula was chosen to correspond most closely with the current consensus definition of sedentary behavior [1], our choice may limit comparisons to previous studies using PPAQ and the originally proposed SED subscale algorithm.

The current study is the first to evaluate the validity of our SB2D questionnaire, asking for separate estimation of non-work and work days and for daily time sitting in hours or on a Likert scale. Assessment of SED duration by the SB2D performed similarly to the PPAQ for ranking of women with *r* and ICCs all below 0.5 and statistical trends in criterion-measured SED across self-report tertiles in only two of the three trimesters. However, the SB2D had greater systematic bias as compared to the PPAQ (3–4 h vs. 1–1.5 h, respectively). Interestingly, SED% from our SB2D instrument resulted in the best agreement with activPAL across the self-report instruments we evaluated. At the same time, however, patterned errors and wide limits of agreement were present. Our findings concur with a recent systematic evaluation of self-report instruments of sedentary time compared to activPAL in older adults [36]. Like ours, this study found self-report instruments had poor validity; however, instruments asking participant to report SED% (via visual analog scale) performed best. Thus, for research where objective monitoring is not feasible, we would recommend our SB2D used herein to measure SED% (see Additional file 1). Reliability of this instrument and testing of further refinements, such as replacing the Likert Scale with a visual analog scale, are areas for future research.

Strengths of our study include the provision of novel validation data for commonly used and newly developed assessment methods for sedentary behavior, across three trimesters of pregnancy, and in comparison to the activPAL monitor that assesses the most current consensus definition of sedentary behavior. Limitations deserving comment include that the women included in this study were adherent, mostly well-educated participants in a longitudinal cohort study; this may have resulted in improved validity and reduced generalizability of our findings. Further, we only evaluated one commonly used reduction algorithm for the Actigraph GT3X and a few self-reported instruments. Though whether agreement would improve or worsen is not certain, it is possible that different accelerometer data reduction procedures (e.g., different cut-points, non-wear rules, use of vector magnitudes) or self-report questionnaires could have yielded different results. Lastly, concurrent wear of the GT3X and activPAL monitors could tend to increase validity estimates, as compared to the PPAQ which queries SED within the current trimester and the SB2D which had no timeframe.

## Conclusions

The findings herein have implications for interpreting existing sedentary behavior in pregnancy research. First, accelerometer-assessed sedentary behavior likely produces reasonable overall mean estimates, but individual errors (while unbiased) can be substantial. Accelerometer- and activPAL-measured sedentary behavior are not interchangeable. Though still preferable to self-report, using accelerometer-measured sedentary behavior will likely yield attenuated associations and measurement errors could limit the ability to detect intervention effects or changes over time. This may be of particular concern for pregnancy research, as changes across pregnancy may be important. The PPAQ sedentary behavior subscale, especially during the later trimesters, should be used with caution. When self-report is the only feasible option, our data suggest that the SB2D querying SED% on work and non-work days yielded the best estimates of sedentary behavior during pregnancy.

## Supplementary information

**Additional file 1.** Sedentary Behavior Two Doman (SB2D) Questionniare. SB2D questionnaire and scoring algorithm.

## Data Availability

The datasets used and analyzed during the current study are available from the corresponding author on reasonable request (Dr. Bethany Barone Gibbs at bbarone@pitt.edu).

## Abbreviations

GPAQ: Global Physical Activity Questionnaire
MoM Health: Monitoring Movement and Health
PPAQ: Pregnancy Physical Activity Questionnaire
SB2D: Sedentary Behavior Two Domain Questionnaire
SED: Sedentary Behavior (hours per day)
SED%: Sedentary Behavior (percent of wear time)
