# Supplementary material for: Validity of self-reported and objectively measured sedentary behavior in pregnancy
Source: BMC Pregnancy Childbirth. 2020 Feb 11;20:99. doi: 10.1186/s12884-020-2771-z (PMC7014698; doi:10.1186/s12884-020-2771-z)
Supplement: Supplementary file 1 — Additional file 1. Sedentary Behavior Two Doman (SB2D) Questionniare. SB2D questionnaire and scoring algorithm. [file 12884_2020_2771_MOESM1_ESM.docx]

**Sedentary Behavior Two Domain (SB2D) Questionnaire**

| The following questions are about sitting or reclining at work/school, at home, getting to and from places, or with friends including time spent sitting at a desk, sitting with friends, travelling in car, bus, train, reading, playing cards or watching television, but do not include time spent sleeping. |
| --- |
| **WORK DAYS**   1. Do you work?   O Yes (answer questions 2 and 3 below)  O No (skip to question 4) |
|  |
| 1. How much do you work?   O Part-time  O Full-time   1. Which of the following best describes the amount of time you spend sitting on a typical **WORK** day?   O almost never  O about ¼ of the time  O about ½ of the time  O about ¾ of the time  O almost all of the time |
|  |

**NON-WORK DAYS**

1. Which of the following best describes the amount of time you spend sitting on a typical **NONWORK** days?

O almost never

O about ¼ of the time

O about ½ of the time

O about ¾ of the time

O almost all of the time

**SCORING**

Answers to questions 3 and 4 are converted to percentages as follows:

O almost never = 5%

O about ¼ of the time = 25%

O about ½ of the time = 50%

O about ¾ of the time = 75%

O almost all of the time = 95%

Individual score is weighted (calculated) as follows:

Nonworking = question 4 percentage

Part-time working = [(2.5/7 days per week) x (question 3 percentage)] + [4.5/7 days per week) x (question 4 percentage)]

Full-time working = [(5/7 days per week) x (question 3 percentage)] + [2/7 days per week) x (question 4 percentage)]
